# “*Vse* (Everyone) Online?”: an exploration of the evolution of the Russian Federation's digital government portal during the COVID-19 pandemic

**DOI:** 10.3389/fsoc.2023.1223957

**Published:** 2023-08-31

**Authors:** Keith Guzik

**Affiliations:** Department of Sociology, College of Liberal Arts and Sciences, University of Colorado Denver, Denver, CO, United States

**Keywords:** digital government, web portal, Russia, legitimacy, COVID-19

## Abstract

The penetration of digital technologies in government has been met with both optimism and caution. This study seeks to contribute to this field by examining *how digital government evolved during the COVID-19 pandemic*. Using media reports on Russia's government services portal (Gosuslugi), it finds that authorities made the portal a centerpiece of their pandemic response by enhancing its *communicative, transactional*, and *participatory* functions. These efforts aimed to not only house public health services on Gosuslugi, but to channel financial, commercial, and communication services through it, expanding Russia's *digital corporatist state*. While pandemic governance infused Gosuslugi with the qualities of a surveillant assemblage, it also made the portal into a space for novel forms of civic participation. Gosuslugi's evolution in this direction was limited, however, by security concerns as well as apprehension about digital participation. These findings highlight the importance of attending to political and cultural contexts in understanding digital government. In Russia, ruling elites' unwillingness to hold competitive elections and the public's lack of confidence in the political system limit the potential of digital government, regardless of its potential to manage crises.

## 1. Introduction

Advanced information and communication technologies (ICTs) inform a growing amount of what government does, from interacting with citizens to procuring supplies, work, and services. Advocates believe digital government will improve the quality and delivery of public services (Jaeger, 2003; The World Bank, 2016; Margetts and Dorobantu, 2019; Twizeyimana and Andersson, 2019), make policy-making more transparent (Keijzer, 2016), and promote civic participation (The World Bank, 2016; Gardels and Berggruen, 2019; Manoharan et al., 2021). More critically minded observers believe state digitalization exacerbates existing structural inequalities (Eubanks, 2018; Benjamin, 2019; Brayne, 2020; Goggin and Soldatic, 2022) and decreases public accountability and the democratic nature of government (Fourcade and Gordon, 2020). While often associated with democratic political systems, digital tools play an important role in authoritarian regimes by enabling the monitoring of civil society and creating channels of civic participation (Yuan et al., 2012; Gunitsky, 2015; Truex, 2017; Toepfl, 2018).

Digital government has been described as “co-evolutionary”, unfolding over time through an interactive process involving engineers, state administrations, users, and technology (Janowski, 2015; Luna-Reyes and Gil Garcia, 2015). Crises, meanwhile, can serve as occasions for states to experiment with new ways of managing public affairs (Wallerstein, 2011; Jessop, 2015). The COVID-19 pandemic, for instance, witnessed the development of mobile tracking applications (Cingolani, 2022), global measures of vaccination coverage (Pichelstorfer and Paul, 2020), and other digital innovations that empowered governments to govern society in new ways (Tazzioli and Stierl, 2021). To date, however, few studies have specifically considered *how digital government evolved during the COVID-19 pandemic*.

This study looks to address this question by examining the development of the Russian Federation's Portal of State and Municipal Services, or Gosuslugi for short, over a 2-year period corresponding with the outbreak of the COVID-19 pandemic. Based on a content analysis of Russian news coverage of the portal, the study finds that Russian authorities made Gosuslugi a central component of its pandemic response by taking advantage of its *communicative, transactional*, and *participatory* functions. These efforts served to not only promote the online migration of government offices and services related to public health, but to channel a broad swathe of financial, commercial, and communication services through the state's digital infrastructure. This reflects the rise of Russia's *digital corporatist state*, where technocratic control over societal decision-making operates through digital infrastructure. While pandemic governance infused Gosuslugi with the qualities of a “surveillant assemblage” (Haggerty and Ericson, 2000) in the service of a “biopolitics” (Foucault, 1976) designed to secure the social body against a deadly virus, it also created novel opportunities for civic participation in Russia's deliberative authoritarian government. Gosuslugi's evolution as a participatory space was limited, however, by *security and privacy concerns* as well as authorities' and citizens' respective *apprehension* of digital participation. These findings confirm those of prior research (Gil Garcia and Flores Zuñiga, 2020) concerning the importance of leadership support and popular trust for successful digital initiatives. More than this, they highlight the importance of attending to political and cultural contexts in understanding the evolution of digital government. In Russia, ruling elites' unwillingness to hold competitive elections and the public's lack of confidence in the political system limit the potential of digital government, regardless of its potential to respond to crises.

## 2. Literature review

### 2.1. Researching digital government

Digital government refers to “the use of information technologies such as the internet to deliver government services and establish relationships between a government and its constituents” (Sriramesh and Rivera-Sánchez, 2006). Various terms are used to refer to this use of ICTs in government, including “e-government” (Coe et al., 2001), “information government” (Mayer-Schönberger and Lazer, 2007) “government 2.0” (Eggers, 2007), “smart government” (Gil Garcia, 2012), and “agile government” (Mergel et al., 2021).^1^ Digital government has been described as “the second revolution in public management after “new public management”” (Saxena, 2005) and is believed to carry a range of benefits. These include more interactive, efficient, and personalized interactions between government agencies and laypeople (Fountain, 2004; Margetts and Dorobantu, 2019), the streamlining of state bureaucracies and operations, heightened professionalism on the part of government employees, improved public service quality (The World Bank, 2016; Twizeyimana and Andersson, 2019), more transparent policy-making and clearer communications with the public (Keijzer, 2016), and the empowerment of citizens through access to services and participation in elections (Henman, 2010; The World Bank, 2016; Gardels and Berggruen, 2019; Manoharan et al., 2021).

A growing body of literature appearing over the last two decades evidences how digital initiatives often fail to meet this potential. The implementation of digital projects, for instance, can be constrained by limited financial resources, the availability of digital technology, dependency on legacy information systems, the reliability of electricity and internet connections, the integration of front-office and back-office information systems, the existence of electronic communication between government units, top management support for initiatives, organizational practices and routines, technological competence among government employees, information security in government units, networks between the public and private sectors allowing for exchanges in expertise, and contextual factors such as laws, regulations, and political systems (Fountain, 2004; Sriramesh and Rivera-Sánchez, 2006; Okunola et al., 2017; Kattel and Mergel, 2018; Gil Garcia and Flores Zuñiga, 2020). The adoption of digital government solutions by citizens, in turn, is impacted by the public's perception of their security and privacy protections, trust in the government, accessibility to digital services through internet and mobile devices, societal capacities for bridging digital divides, the quality of the information and services provided by the state, people's awareness of these services, and personality type (Venkatesh et al., 2014; Luna-Reyes and Gil Garcia, 2015; Gil Garcia and Flores Zuñiga, 2020; Cingolani, 2022). Digital government can often become lodged at simpler stages of development, such as the dissemination of information about government services, rather than advancing to more advanced stages, such as integrated service delivery and civic participation (Holliday and Kwok, 2004; Torres et al., 2006; The World Bank, 2016).

More recent research in critical algorithm studies and related areas has also highlighted the risks of digital government. ICTs have been found to widen digital divides along age and vocational lines (Lee and Cho, 2007), produce new forms of digital exclusion for those differently abled (Goggin and Soldatic, 2022) or deserving of state benefits (Eubanks, 2018; Schou and Pors, 2019), and reinforce established structural inequalities along race and class lines (Benjamin, 2019; Brayne, 2020). Intersectionality between age, race, and location can compound disadvantage (Sourbati, 2009; Rosenberg, 2021; Goggin and Soldatic, 2022). Advanced technologies also bring about “new entanglements between the military and actors in the corporate domain” (Hoijtink and Hardeveld, 2022). On the basis of such findings, Fourcade and Gordon (2020) have described the emergence of the “dataist state” as a new mode of governance that enables “algorithmic violence” (Bellanova et al., 2021) and is less accountable, less humane, and more corporate than its predecessors (see also Madsen et al., 2016).

### 2.2. Digital government in authoritarian regimes

The easy transmission of information afforded by networked digital technologies disrupts the state's historic balance of power, especially for authoritarian regimes operating on tightened social control (Oates, 2013; Owen, 2015). Authoritarian governments have responded to this threat by using technological and legal mechanisms to block access to restricted websites (Barmé and Ye, 1997), limit online content (Shevtsova, 2015), and seize majority interest of leading technology companies (Budnitsky and Jia, 2018). At the same time, the relationship between digital technologies and authoritarian government involves more than the interplay of technological freedom and state repression. Digital technologies allow authoritarian regimes new ways to legitimate their rule (Johnson and Kolko, 2010). Social networks, for instance, can be leveraged to mobilize pro-regime support, frame public debate, and manage public outrage (Toepfl, 2011; Gunitsky, 2015). Authoritarian governments can also launch initiatives to increase people's access to the Internet and government and financial services, which can increase citizen satisfaction with the government (Yuan et al., 2012; Truex, 2017).

Also, not all authoritarian regimes are alike (Levitsky and Way, 2010). Non-competitive authoritarian regimes have been found to create a governmental web presence primarily for international rankings, while competitive regimes possess digital facilities similar to those of democracies (Maerz, 2016). “Consultative” (Truex, 2017) or “deliberative” authoritarian regimes (He and Warren, 2011) create and rely on “input institutions” (Nathan, 2003) that invite citizens to monitor certain policies or lower-level officials, discuss planned government measures, and aggregate citizen preferences online (Jiang and Xu, 2009; Gunitsky, 2015; Truex, 2017; Toepfl, 2018). Citizens in deliberative authoritarian states like Russia operate in an information environment with considerable choice (Robertson, 2015), and political opinions regarding the government are more varied than usually thought (Robertson, 2015).

### 2.3. The co-evolution of digital government and states

The preceding points demonstrate how digital government is shaped by internal and external contexts. It unfolds over time through a complex, recursive process involving private developers, government units, state administrations, work practices, technological software and hardware, legislation, and users (Luna-Reyes and Gil Garcia, 2015). This “co-evolutionary” (Luna-Reyes and Gil Garcia, 2015) perspective resonates with a broader view of states and societies as historical objects (Migdal and Schlichte, 2005). Seminal works in state formation and political philosophy demonstrate that social crises such as wars, famines, pandemics, and economic collapses serve as particularly salient occasions for states to experiment with new ways of managing their affairs—the prison (Foucault, 1977), mass digital surveillance (Lyon, 2003), group therapy (Miller and Rose, 1988), and other government technologies have their origins in such crises.

Contemporary society, for its part, has been described as in a state of “perpetual crisis” (see also Wallerstein, 2011; Beckett, 2019), beset with natural disasters, wars, organized crime, terrorist attacks, pandemics, and economic decline. These calamities can be expected to impact the evolution of digital government. The COVID-19 pandemic, for instance, “opened up many opportunities for crisis learning” (Mueller, 2022) and witnessed the development of new tools—mobile tracking applications (Cingolani, 2022), restrictive immigration rules (Tazzioli and Stierl, 2021), and global measures of vaccination coverage (Pichelstorfer and Paul, 2020)—that rationalize and manage society in new ways. To date, however, few studies have sought to explore the broader evolution of digital government during the pandemic. This study looks to fill this gap by examining *how digital government evolved during the COVID-19 pandemic*.

## 3. Methods

To address this question, this study employs a single case study methodology to examine the Russian Federation's Unified Portal of Government and Municipal Services (*Yedinyy portal gosudarstvennykh i munitsipal'nykh*), more commonly referred to by the abbreviation Gosuslugi (*Gosudarstvennoe Uslugi* in Russian, or *State Services* in English), over a two-year period, starting with the onset of the COVID-19 pandemic in March, 2020 and ending in February, 2022.^2^ While conceptions of case studies differ widely (see Ragin and Becker, 1992), one commonly accepted definition is “an empirical inquiry that investigates a contemporary phenomenon within its real-life context” (Yin, 2003). Case studies are an appropriate methodological choice when research questions are “exploratory” in nature (Yin, 2003), which is the instance here in studying the evolution of digital government during a social crisis.

A single-case design, for its part, is an accepted methodological choice when the case in question is “representative or typical” (Yin, 2003) or belongs to a specific family of phenomena (Walton, 1992). Gosuslugi fits this criterion. Web portals are a prime example of digital government. They serve as “one-stop shops” providing citizens and businesses information about government operations, access to state services, and the opportunity to participate in government decision-making (Gant and Gant, 2002; The World Bank, 2016). Gosuslugi is the Russian Federation's national portal.^3^ It was launched in 2009 as part of the Russian Federation's “Electronic Russia” initiative (2002–2010) seeking to strengthen economic efficiency, information dissemination, democratization, and ICT infrastructure, and it matured during the government's “Information Society” (2011–2020) initiative aimed at media environment diversification, cybersecurity, and e-government (Zherebtsov, 2019; Gritsenko and Zherebtsov, 2021).^4^ The portal is administered by the Ministry of Digital Development, Communications and Mass Media (*Ministerstvo tsifrovogo razvitiya, svyazi i massovykh kommunikatsiy Rossiyskoy Federatsii*), or MinTsifry.

Previous research has highlighted Gosuslugi as a notable achievement in the Russian government's efforts to digitize the state (Zherebtsov, 2019). Supported by the Integrated System of Identification and Authentication (*Yedinoy sisteme identifikatsii i autentifikatsii*), or ESIA, which verifies users' identity online across state-level and federal-level agencies, Gosuslugi offers Russian a wide range of government services and information online. These include paying traffic fines, renewing car registrations, enrolling children in schools and daycare, and signing up for doctor's visits at state clinics. The portal has a high level of reported user satisfaction (Zherebtsov, 2019), and its success helped propel Russia up global indices of digital government. In 2010, the country ranked 59th in the UN's e-government development index (EGDI), whereas in 2020, it ranked 36th (United Nations, 2022, see also Gerasukova, 2020).

The COVID-19 pandemic, meanwhile, represented a genuine crisis for the Russian Federation, as it did for much of the world. Upon the arrival of the virus's first wave in March 2020, republics and municipalities across Russia implemented a quarantine regime that required individuals to stay at home except in cases of medical emergencies or to go to the grocery store, travel to work if required, take out the trash, or walk a pet (Wikipedia, 2022). More relaxed quarantine regimes were instituted for subsequent waves of the pandemic in September 2020, July 2021, and October 2021. Russia developed one of the world's first vaccines, Sputnik V, which became available to the public in December, 2020 (Oxenstierna, 2021). But vaccination rates in the country lagged behind those of its Western and Eastern counterparts (Ritchie et al., 2020). Covid infection and death rates in Russia, meanwhile, were higher than in other countries (Ivanova et al., 2021). In brief, the pandemic disrupted various dimensions of social life across the time of this study. As such, it serves as an appropriate period for examining the development of digital government during a social crisis.

### 3.1. Data and analysis

To examine the evolution of Gosuslugi during the pandemic, this study relies on a qualitative content analysis of primarily Russian-language news reporting about the portal. The news items were gathered through a weekly Google Alerts service, which amassed material from national, regional, local, and online news outlets in Russia. The news items were maintained in a single Microsoft Word file. Ultimately, the file contained 587 separate items consisting of 189,310 words.

This material was analyzed using a hybrid grounded theory approach (Corbin and Strauss, 2014). The author, together with a native Russian-speaking undergraduate student, coded the database both inductively and deductively through two rounds of coding. In the first round, we identified latent and manifest themes inductively, while also applying deductive codes drawn from the digital government literature to identify functions of the portal (*information, transactions, transparency, engagement, integration*, and *security*) (Luna-Reyes and Gil Garcia, 2015). In the second round of coding, we synthesized the initial codes to increase parsimoniousness and decrease repetition.

These methods are not without limitations. Media coverage is not the most reliable data source, given the political and financial concerns that inform coverage (Rubin and Babbie, 2007). This is especially important to consider in Russia, where the government restricts the press (Shevtsova, 2015; Szostek, 2018). Thus, while all news media is subject to reporting bias (Yin, 2003), that in Russia is prone to coverage favorable to the state and/or unlikely to draw rebuke from state authorities. These limitations notwithstanding, reliance on news items has been successfully utilized in previous studies of digital government in Russia (see Toepfl, 2011, 2018). In addition, Russian news media during the time of the study was fairly diverse, consisting of both elite-controlled official media and liberal, oppositional media (see Toepfl, 2018). This work draws from both spheres. Finally, this is an exploratory study looking to understand general trends in the portal's development rather than the meanings it holds for the government or citizens. In this sense, news reporting provides an adequate, if imperfect, view of the portal for the purposes of discerning its recent history.

## 4. Findings

The analysis of news items related to Gosuslugi over the first 2 years of the COVID-19 pandemic revealed a number of themes. Unsurprisingly, the one dominating news reporting was the pandemic. The existence of the portal as a hub for public services made it a critical tool supporting the government's enforcement of quarantines, promotion of social distancing, and distribution of goods and services to counter the virus. But not all elements of Gosuslugi's development over this time related to public health. To make sense of these developments, this section borrows from past research describing portals in terms of their functionality (Jayashree and Marthandan, 2010; Yuan et al., 2012; Luna-Reyes and Gil Garcia, 2015). As the following sub-sections explain, during the COVID-19 crisis, Russian authorities worked to enhance the *communicative, transactional*, and *participatory* capacities of Gosuslugi in order to manage both the virus and social life more generally.

### 4.1. Communication

Communication is the simplest function of portals, involving the unidirectional transmission of information from the government to users (Jayashree and Marthandan, 2010). During the pandemic, the capacity of Gosuslugi to share information was utilized by government officials to publicize information about a highly transmissible and dangerous disease about which society initially knew little.

Two types of communication were commonly noted in the news reporting. The first was *webposts*, or making information about the virus or the government's efforts to contain it accessible to the public on the Gosuslugi site. For instance, in March 2020, when the pandemic began in Russia, the government created a dedicated webpage—*Vse.Online* (Everyone Online)—sharing information about the illness, regulations such as the quarantine for controlling its spread, and links to public and private services to help people cope with the quarantine regime (these included Sberbank's telemedicine service, DocDoc; food delivery applications like DeliveryClub and Yandex.Lavka; and online video streaming services, such as ivi and Okko). *Vse.Online* was housed within and highlighted on the main Gosuslugi site, presenting users visiting the portal with the opportunity to access the page. The second form of communication was *messaging*. For registered users of Gosuslugi, portal administrators sent direct messages about the importance of observing the quarantine and social distancing rules (Sukhorukov, 2020) and the availability of services to counteract the virus, such as testing or vaccines.^5^

Comparing these two forms of communication, messaging provides advantages over webposts. Authorities can use messaging to deliver information directly to an individual user and see whether the information has been accessed. But with both messaging and webposts, the government is unable to determine to whether the information has actually been read and comprehended.

### 4.2. Transactions

Transactions involve a higher level of functionality, which requires support for two-way interactions between government workers and citizens in an online environment (Jayashree and Marthandan, 2010). Transactional functionality allows for such operations as submitting forms for the provision of state services or completing civic obligations like paying taxes. Most of the news coverage on Gosuslugi during the pandemic touched upon on its transactional dimensions. These transactions can be categorized by purpose: the *provision of social benefits*, the *administration of public health measures*, and the *facilitation of private commercial and financial services*.

The Russian Federation, like governments across the world, implemented a range of *social benefits* to offset the financial impact of the pandemic and quarantine measures. Families, for instance, were eligible to receive a one-time outlay from the federal government of 10,000 rubles (roughly 150 dollars) for each child between 3 and 16 years old. Gosuslugi served as the primary means for delivering the benefit. Within the first days of the program, 2 million families applied for the benefit through the portal (Alpatova, 2020). Existing state benefits, meanwhile, were also moved online and made available via the portal to accommodate social distancing rules. Those displaced from their jobs because of illness and workplace closures were able to access unemployment benefits if they were authenticated Gosuslugi users (Rossiyskaya Gazeta, 2020a). Pension payments similarly were made available through the portal during this time (Rossiyskaya Gazeta, 2020a).

Transactions related to the *administration of public health measures* received the most coverage in the news sample. These included a mixture of both old and new measures. For example, when Russians fall ill, they require sick leave (*bolnichnii list*) to be excused from work. As the first wave of COVID-19 spread across the country, authorities sought to allow for the continued operation of the sick leave system without flooding medical offices with individuals infected with the virus. Thus, Gosuslugi users could receive *bolnichnii lists* upon request through the portal (Dvinanews, 2020).

With regard to newer health measures, MinTsifri, Gosuslugi's governing authority, launched the “Gosuslugi STOP Coronavirus” application for creating digital passes based on QR codes to permit travel during the quarantines (Klokov, 2020). The Gosuslugi portal gained additional functionality with the development of coronavirus tests and vaccines. Russians starting from January 2021 were able to sign up to receive the Sputnik V vaccine at both state and private clinics through the Gosuslugi site (Vedomosti, 2020). When subsequent waves of the pandemic arrived, authorities folded testing and vaccine requirements into quarantine rules. Certain public spaces, such as theaters and museums, would remain open, but only to those with QR codes demonstrating proof of vaccination, having recovered from infection, or a negative test result. Public and private clinics provided data on those receiving tests and vaccines directly to the government, which would then send and store QR codes in the personal accounts of Gosuslugi users (Meduza, 2021).

MinTsifri also worked to have Gosuslugi serve the *facilitation of remote commercial and financial services*. In May 2020, MinTsifri, together with the Central Bank of Russia, announced a pilot project that would allow Russians to apply for credit, mortgages, and insurance from banks and insurance companies through the portal. The service would increase the efficiency and security of financial operations by allowing companies to receive personal data about potential borrowers and clients housed in different government databases (for instance, the Federal Tax Service, the Pension Fund, etc.). Similarly, in June 2020, MinTsifri announced a new project to authenticate the identity of users on digital marketplace websites. These sites included Avito, a peer-to-peer digital marketplace; Yandex, an online retailer; Tsian, a real estate website; and Avto, a car sales website (Tishina, 2020). The identity authentication service would reduce both fraud and administrative costs for these sites by eliminating the need to verify user identity through private commercial services and gather documents such as car titles and property deeds from users themselves (Zadorozhniy, 2020).

### 4.3. Participation

News coverage during the first 2 years of the COVID-19 pandemic also highlighted how MinTsifri sought to make the Gosuslugi portal a locus for the increased participation of Russians in the country's public affairs. Participation represents a higher-level function for government portals, requiring a technical capacity for two-way expression and a responsiveness on the part of authorities to citizens (Jayashree and Marthandan, 2010). With Gosuslugi, news reporting noted two general directions for participation. The first was in service of the government's *public health measures*, while the second was in support of *political elections*.

Regarding public health measures, early in the pandemic, federal authorities used the portal to send out a call to users inviting them to sign up as volunteers to assist elderly persons who were self-isolating because of the coronavirus epidemic (Meduza, 2020). The government also sought users' feedback on service quality. For example, doctors could lodge complaints about difficulties receiving additional compensation for working with covid-19 patients (Petrova, 2020). Laypeople, meanwhile, were invited to share problems with having doctors and nurses make home visits to deliver care (Protsenko, 2020).

Turning to *politics*, the government's desire to promote social distancing during the pandemic saw it expand Gosuslugi's functionality in political elections. One way it did this was by using the portal as a means for potential candidates to *collect signatures for registering their campaigns*. Three regions—Chuvashia, Permskii Krai, and Chyelyabinskaya—participated in pilot projects where candidates could gather electronic signatures through the Gosuslugi site. The types of office and the number of signatures one could collect were limited in each case. Chuvashia and Persmkii Krai allowed the collection of digital signatures for gubernatorial candidates, while Chyelyabinskaya allowed it for regional parliamentary deputies (Ponomarev, 2020). In each case, no more than half of the signatures collected could be electronic (TASS, 2020).

Gosuslugi was also utilized during the pandemic for the *casting of votes in national elections*. From June 25 to July 1, 2020, Russia held a national vote on amendments to its constitution allowing, among other things, President Vladimir Putin to run for office a fifth and six time in 2024 and 2030 (Tack and Hawn, 2021). The government was eager for citizens to participate in the referendum to build support and increase the legitimacy of the amendments. To that end, MinTsifri announced that those interested in voting remotely through the Gosuslugi portal would be able to do so (Fontanka, 2020). In the September 2021 parliamentary elections, Russians in certain regions—Moscow, Sevastopol, Kursk, Murmansk, Nizhne Novgorod, Rostov, Yaroslav—also had the opportunity to cast their votes remotely using Gosuslugi (Stashchenko, 2021). Finally, in March 2022, President Putin signed into law amendments adopting the remote electronic voting system across the country (The Moscow Times, 2022).

### 4.4. Successes and challenges

Although the news reporting analyzed in this study was descriptive rather than evaluative, it did touch upon the outcomes of the government's efforts to expand Gosuslugi. These outcomes can be classified into *successes* and *challenges*. Beginning with *successes*, the news items noted increased usage of the Gosuslugi site during the time of the pandemic. During the first months of the pandemic, the number of citizens using online government services almost doubled, totaling 51% of the Russian population (Rossiyskaya Gazeta, 2020b). In addition to increased usage, how Russians used the portal also changed. Before the pandemic, the most popular services on Gosuslugi were making a doctor's appointments, enrolling in daycare, registering an automobile, and receiving driver's licenses and passports. During the pandemic, that list still included making a doctor's appointment, but it now included applying for the one-time benefit for children from 3 to 16 years old, registering for sick leave, and registering for unemployment benefits (Alpatova, 2020).

The challenges associated with Gosuslugi during this time can be categorized into three types: *technical, security*, and *distrust*. Government efforts to increase the functionality of the Gosuslugi portal to accommodate the government's response to COVID-19 repeatedly met with diverse types of *technical challenges*. None of these seriously compromised the operationality of the portal. But they disrupted its operation on a short-term basis, leading to frustration by users. For instance, the portal would often become inaccessible when usage surged, as occurred when it began accepting applications for the one-time family benefit and when the site opened for vaccination applications (Bakhmutskaya, 2021).

*Security issues* received considerable attention in the press. These related to the *vulnerability of the portal* to hacks and data leaks and the *vulnerability of individual users* to hacks and fraud. Gosuslugi usernames and passwords that could be used to access users' accounts were found for sale on darkweb sites for 20 rubles (or about $0.30) per listing (Roskomsvoboda, 2021). Attacks on the Gosuslugi site also sought to disable its functionality. For instance, in May 2021, during the runup to the parliamentary elections in September, various Gosuslugi users discovered their personal data had been modified in the portal and that they had been registered to participate in the primaries of the United Russia party, the dominant ruling party in Russia. One of the first people to discover this hack was Arina Borodina, a journalist for Echo Moskva, an independent radio station known for its criticism of the government (Kommersant, 2021). United Russia, for its part, verified that it had recorded numerous attempts by users with stolen accounts to register in their primaries and considered the affair an effort to delegitimize remote voting through the Gosuslugi site (Zhukova and Yuzbeka, 2021).

Stories of *individual Gosuslugi users targeted by cyberhackers and cyberfraudsters* also appeared frequently in the news. Attacks on individual users commonly had the goal of either directly *defrauding them* or *acquiring their personal data* to assume their identity (Izvestia, 2020). In the first instance, fraudsters might send Gosuslugi users an announcement about extending their car insurance coverage or paying off a traffic fine online, together with data about their car and registration and links for making payment that would deposit the funds in an account accessible to the criminals (Ilina, 2021). With regards to the second type, fraudsters posing as Gosuslugi employees would send potential victims an email notifying them that their Gosuslugi accounts had been hacked and asking them for their personal data to verify their identity and reactivate the accounts. With that data, the schemers could then take out loans in the victims' name via the Gosuslugi site (Maksimova, 2021).

Finally, the government's efforts to extend the functionality of Gosuslugi into politics was limited by *distrust* in the portal. In the 2021 parliamentary elections, opposition parties reported problems registering their candidates for office, which they attributed to Kremlin concerns with rising popular dissatisfaction in the country (Nezavisimaya, 2020). During the election itself, United Russia won the most legislative seats in the Moscow region based in part on its strong performance among ballots cast online. However, the count of ballots was delayed for days, during which time five opposition party candidates saw their vote leads erased. The delay was officially attributed to encrypted votes being tabulated (Venkina, 2021). But the explanation did little to assuage the suspicions of critics that the digital vote was rigged.

## 5. Discussion

The findings presented above demonstrate some of the ways in which the communicative, transactional, and participatory functions of the Russian Federation's Gosuslugi government services portal evolved in order to provide Russian citizens information about the virus and the government's response to it, access to government services and benefits, medical resources, and travel permits meant to minimize the virus, and novel opportunities to access commercial and financial services and participate in civic life. Through these efforts, ordinary Russians' relationship to the portal was modified, as the number of users using the portal and the types of activities they completed changed. But the potential of Gosuslugi to serve as a transactional and participatory hub of Russian life was also limited by technical challenges, security concerns, and apprehension.

This study is clearly not without limitations. For one, Gosuslugi is a single government services portal, while digital government represents a much larger phenomenon. To better understand the evolution of digital government during times of crisis, additional portals (whether municipal or regional portals in Russia or national portals in other countries), types of digital initiatives (digital identity initiatives, decision-making supported by Big Data analytics, etc.), and varieties of crises (wars, climate change, cultural conflicts) could be studied comparatively. Second, as noted before, the study relies on news reporting of Gosuslugi, which presents validity concerns. To bolster confidence in these findings, additional types of data—interviews with government officials or employees managing or working with Gosuslugi or ordinary Russians using the portal—would be needed to triangulate the data.

Nevertheless, the present case study presents meaningful findings on the development of digital government in times of social disruption. The following discussion section considers four themes—the evolution of digital government in terms of transactional and participatory functionality, the reemergence of the authoritarian corporatist state in digital society, the surveillant and participatory tendencies of digital government, and the role of legitimacy in shaping the boundaries of digital government.

### 5.1. The transactive and participatory evolution of Gosuslugi

Prior research notes how governments often face difficulties moving digital initiatives from informational to transactional and participatory stages (Holliday and Kwok, 2004; Torres et al., 2006; The World Bank, 2016). Researchers have offered more subdued assessments of Gosuslugi, in particular, based on its primary function being informational (Gritsenko and Zherebtsov, 2021) and for having a low number of authenticated users (Zherebtsov, 2019). Findings from this study suggest that, during a time of crisis, MinTsifri was able to increase the number of Gosuslugi users and increase the transactional and participatory functionality of the portal.

The motivation behind this shift seems clear. During the COVID-19 crisis and accompanying quarantines, the government offered citizens various incentives—QR codes providing freedom of movement, vaccinations, and medical visits to protect their health as well as economic payments to support their families—allowing them to manage the physical, mental, and financial risks in their lives. Gosuslugi served as the means through which people could access those benefits. In this sense, Russian authorities were able to evolve the portal by making it an “obligatory passage point” (Callon, 1984) for Russians wishing to access resources protecting them against the health crisis and a fuller range of rights and privileges restricted by the quarantines.

This finding, in turn, has implications for understanding the evolution of digital government. In the digital age, states facing “growing temporal pressures in policy-making and implementation due to new forms of time–space distantiation” have been found to respond to “to economic events, shocks and crises” by “withdrawal from areas where states are actually or allegedly too slow to make a difference” or by “shortening of policy development cycles” (Jessop, 2015). COVID-19 represents a crisis whose rapidity would seem to challenge traditional capacities of the state. However, Russian authorities were able to leverage the existing digital infrastructure of the state—Gosuslugi—to quicken their response to the crisis. And in doing so, they enhanced what Cingolani (2022) refers to as the state's “digital infrastructural power,” through which political elites are able to extract resources from and provide services to political subjects. This demonstrates how crises can serve as generative moments for digital government as they have for other types of governmental innovations in the past.

### 5.2. Gosuslugi and the evolution of Russia's digital corporatist state

Gosuslugi's status as the Russian Federation's national government services portal made it a natural answer to the question of how to direct central elements of the COVID-19 pandemic response—information campaigns, provision of social welfare benefits, issuance of QR codes and vaccination certificates, signing up for vaccinations, and so forth. Interestingly, however, the Russian government incorporated not only public health services into the Gosuslugi ecosystem, but existing private sector online services as well—bank loans, car insurance, online marketplaces. These findings evidence the evolving “trajectory” (Migdal and Schlichte, 2005) of what can be referred to as Russia's *digital corporatist state*. As with previous forms of “corporatism,” “societal decision-making is almost exclusively in the hands of technocrats so as to reduce or avoid open and violent conflict between the different groups of interests” (Molina and Rhodes, 2002; Sriramesh and Rivera-Sánchez, 2006). But that governance now operates through the state's digital infrastructure.

The rise of the digital corporatist state has been noted in studies of China, Russia, and Singapore (Sriramesh and Rivera-Sánchez, 2006; Yuan et al., 2012; Ryzhkov, 2014). In response to the 2011–12 anti-government protests, Russian authorities took various measures in this direction, such as forcing leading Internet company, Yandex, to restructure its corporate board to give state representatives a controlling interest and making strategic investments across public sectors (Budnitsky and Jia, 2018; Zherebtsov and Goussev, 2020). The digital corporatist state also fulfills a vow by incoming President Vladimir Putin in a 1999 meeting with IT sector leaders to reign in Russia's Internet after a decade of free development (Gritsenko and Zherebtsov, 2021).

But this is not only a story of governmental control. Corporatism involves a balance of interests. Having Gosuslugi authenticate users' identities in the digital marketplace or for bank loans saves companies money by removing this expense from their operations, and it also provides the state access to data on these financial transactions, which it can monetize on data markets (Kucher, 2020). This speaks to the “dynamics of valuation” at play with digital government (Madsen et al., 2016). Private companies in Russia have also shown a willingness to censor anti-regime messages (Baezer et al., 2021) and to engage in voter intimidation (Frye et al., 2019), and they profit when businessperson candidates win municipal and regional offices (Szakonyi, 2020). This represents a form of “substitutional delegation” (Hedberg, 2016), where the Russian state relies on the private sector to carry out public functions. In brief, the COVID-19 crisis served as an opportunity for the federal government and leading financial and technology companies to deepen their ties in governing Russian society through Gosuslugi's digital architecture.

### 5.3. Gosuslugi as surveillant assemblage

The place of Gosuslugi in Russia's digital corporatist state owes largely to its ability to authenticate personal identity through the Unified System of Identification and Authentication, or ESIA. The findings from the present study confirm, however, that personal identity in an online environment is not a static construct, but an evolving one (Lupton, 2019). The Russian Federation's work to intervene against the COVID-19 pandemic, as well as its efforts to harness private online entities, added new layers to citizens' digital identities. Vaccination status, COVID test results, parental status, pension eligibility, credit worthiness, political party affiliation, and other traits all make up Russians' digital profiles on the Gosuslugi portal. What's more, these traits served as markers enabling or restricting opportunities to participate in society. This was most starkly demonstrated during quarantines, when vaccination status, COVID tests, and occupation type granted one authorization to travel and access public spaces.

Gosuslugi's operation in this regard displays the qualities of a “surveillant assemblage” (Haggerty and Ericson, 2000). The fact that the Russian Federation sought to use this assemblage to limit the spread of a deadly virus and to protect public health through quarantines, social distancing, and the provision of medicines and vaccination infused a “biopolitics” into the portal as well (Foucault, 1976). These findings echo past research demonstrating how crises, including COVID-19 (Tazzioli and Stierl, 2021), enhance state power over individuals and support critical interpretations of digital government as more corporate and less humane than its predecessors (Fourcade and Gordon, 2020).

But this operation of Gosuslugi cannot be divorced from other dimensions of the portal that sought to create new opportunities for participating in Russian civil society. Surveillance offers both “care” and “control” (Lyon, 2001). And MinTsifri and other authorities in Russia worked to have Gosuslugi serve as a medium for Russians to provide feedback on the quality of care they received during the pandemic, to report their health status, to receive state benefits, to participate in political elections, to apply for credit more easily, and so forth. Gosuslugi also provided care by securitizing transactions in the digital marketplace, where risk increases in the absence of the traditional “tokens of trust” present in physical social interactions (Lyon, 2001). For Russians forced into cyberspace for the first time due to quarantines, lockdowns, and social distancing, the portal allowed them to maintain their connection to critical public and private services.

### 5.4. Gosuslugi and the participatory limits of digital government in authoritarian regimes

Digital government initiatives can serve a critical function for consultative authoritarian regimes by creating “input” channels for collecting information about society, coopting elite and oppositional elements in society, and legitimizing the government to the general population (Toepfl, 2018). Prior studies have shown the Russian government to be more open to political competition (Toepfl, 2018; Szakonyi, 2022) and more responsive to citizens' concerns (Maerz, 2016) than other authoritarian governments. The Russian government's attempts to enhance Gosuslugi's participatory capacity by inviting medical professionals and laypeople to report problems with health services, allowing political candidates to collect signatures supporting their candidacies, and hosting elections online are, thus, consistent with past research.

But prior research has also noted how digital government in Russia has been stunted by “the country's vertically integrated political processes” (Zherebtsov, 2019). Limiting the number of electronic signatures a potential candidate can collect through Gosuslugi represents a form of “candidate filtering,” a common tactic of electoral manipulation in Russia (Szakonyi, 2022). And it demonstrates the unwillingness of the regime to permit competitive elections at the national level (Ross, 2018). The skepticism of political parties and the public to digital voting, meanwhile, demonstrates how deficits in institutional legitimacy in Russia (Hutcheson and Petersson, 2016) can serve as an obstacle in the government's plans to foster participation through digital initiatives. In authoritarian countries like Russia, then, a tension is present between government leaders supporting digital initiatives to increase civic participation while ensuring that such participation does not threaten elites' hold on power (Toepfl, 2018; Gritsenko and Zherebtsov, 2021). These findings suggest a limit to both the participatory potential of digital government in authoritarian regimes and the ability of these regimes to legitimate their rule through digital means.

Each of these points of discussion warrants greater attention in future research. In-depth interviews and social surveys could provide insight into how ordinary Russians experience transacting with and participating in Gosuslugi and how private institutions, organizations, and individuals interpret and react to the increasing power of the Russian digital corporatist state. In-depth interviews with individual Russians would also help better understand the balance between the controlling and caring dimensions of the Gosuslugi surveillant assemblage. Comparative studies of digital portals in democratic and authoritarian countries, meanwhile, would allow for a deeper appreciation of how political contexts can shape digital government in particular directions. Finally, it is important to remain attentive to how emergent crises–Russia's war in Ukraine and the continued fracturing of the global order—might further influence the evolution of digital government in the future.

## 6. Conclusion

The increasing penetration of digital technologies into the organization and operation of government has been treated with both optimism and caution by researchers. Bridging the distinct interpretations of digital government is an evolutionary perspective that understands both the state and technology as a social construction. This study has sought to contribute to that understanding by studying the development of digital government during a time of crisis, the ongoing COVID-19 pandemic. Using the Russian Federation's government services portal as a case study, and relying on media reports on its development over the first 2 years of the pandemic, the study finds that Russian authorities made the digital portal a centerpiece of their pandemic response by enhancing its *communicative, transactional*, and *participatory* functionality. These efforts aimed to not only migrate government offices and services related to public health online, but to channel a broad swath of financial, commercial, and communication services through the state's digital infrastructure. These efforts increased the number of Gosuslugi users and enhanced the portal's *transactional* and *participatory* functionality. While pandemic governance infused Gosuslugi with the qualities of a “surveillant assemblage” (Haggerty and Ericson, 2000) in the service of a “biopolitics” (Foucault, 1976) intended to secure the social body against a deadly virus, it also made the portal into a space for novel forms of civic participation. Gosuslugi's evolution in this direction was limited, however, by security and privacy concerns as well as authorities' and citizens' respective apprehension of digital participation. These findings highlight the importance of attending to political and cultural contexts in understanding the evolution of digital government. In Russia, ruling authorities' unwillingness to cede their political control to competitive elections and the public's lack of confidence in the political system limit the potential of digital government, regardless of its potential to respond to crises.

## Author contributions

The author confirms being the sole contributor of this work and has approved it for publication.

## Footnotes

^1^These terms are not synonymous. E-governance, for instance, refers to the role of digital technology in the interaction of government, public service, and citizens in the political process, while e-government refers more narrowly to online service delivery and the transformation of public administration through ICTs (Torres et al., 2006). Nonetheless, they each refer to the general relationship between digital technology and the management of public affairs in society.

^2^The end of February 2022 brought a new disruption to Russia in the form of its military invasion of Ukraine and the resulting economic sanctions from Western and Asian democratic countries.

^3^Regions and municipalities in Russia possess their own service portals. The lack of a single integrated, synchronized government services portal has been taken as an indication of the failure of public sector reform in the country (see Gritsenko and Zherebtsov, 2021).

^4^The digitization of the public sphere in Russia occurred simultaneous to reforming the public sector (Gritsenko and Zherebtsov, 2021). The reform efforts have been deemed unsatisfactory (Zherebtsov, 2019). As a result, digitization simply replaced or occurred on top of the pre-existing administrative infrastructure rather than rebuilding or transforming it (Zherebtsov, 2019; Marina and Putintseva, 2020).

^5^Registered Gosuslugi accounts are differentiated by whether they have been authenticated or not. Authenticated accounts signify that the user's identity has been authenticated and they can access all the services that exist in electronic form. Nonauthenticated accounts provide only a limited number of services. The portal has been criticized in the past for inflating its reported level of adoption by using the higher number of nonauthenticated accounts rather than authenticated accounts (Zherebtsov, 2019).
